# Editorial: Advancements in neural coding: sensory perception and multiplexed encoding strategies

**DOI:** 10.3389/fncom.2026.1834521

**Published:** 2026-04-08

**Authors:** Mohammad Amin Kamaleddin

**Affiliations:** Temerty Centre for Artificial Intelligence Research and Education in Medicine, University of Toronto, Toronto, ON, Canada

**Keywords:** context-dependent representation, information processing, multiplexing, neural coding, neural networks, neuromorphic computation, sensation

Understanding how the nervous system encodes and interprets sensory information remains a central challenge in neuroscience. Classical models have often conceptualized sensory pathways as hierarchical and largely feedforward systems that relay stimulus features with increasing abstraction (Felleman and Van Essen, 1991; Riesenhuber and Poggio, 1999). However, accumulating evidence suggests that neural coding is far more dynamic, context-dependent, and multiplexed than previously assumed (Kamaleddin Ezabadi, 2022; Kamaleddin et al., 2021; Kayser et al., 2009; Panzeri et al., 2010). The contributions gathered in this Research Topic collectively advance this perspective by integrating theoretical, computational, and applied approaches to sensory coding across biological and artificial systems.

A key conceptual contribution is the notion that sensory signals are inherently context-dependent representations rather than fixed encodings. The perspective by Ethier et al. introduces the concept of sensory polysemia, proposing that identical neural activity patterns can convey different meanings depending on behavioral, emotional, hormonal, and motivational states. Drawing on the trigeminal system, the authors show how inhibitory circuits gate sensory transmission at early stages. This enables flexible interpretation of tactile inputs. This framework challenges strictly feedforward accounts of perception and instead emphasizes that meaning is constructed through interactions between incoming stimuli and internal state. Such a view aligns with broader theories of predictive and active sensing, in which perception is shaped by expectations and task demands (Clark, 2013; Friston, 2010).

Complementing this conceptual advance, several contributions explore mechanistic and computational implementations of multiplexed encoding. Yedutenko et al. present a detailed investigation of time-difference encoders (TDEs) in spiking neural networks, demonstrating how motion information can be encoded through temporal correlations in event-based sensory streams. Their introduction of a three-point architecture (TDE-3), incorporating inhibitory input, addresses a key limitation of earlier models by restoring direction selectivity in complex environments. This work further highlights that inhibitory interactions are not merely suppressive but play an active computational role in disambiguating sensory signals, echoing biological circuit principles (Isaacson and Scanziani, 2011). The study also illustrates how velocity information can be multiplexed across spike count and inter-spike interval coding schemes, revealing trade-offs between robustness, precision, and latency in neural representations.

At a broader theoretical level, Cariani and Baker provide a comprehensive framework for understanding neural coding by emphasizing the role of temporal structure in sensory representations. Their work systematically surveys different classes of neural codes, including rate-based, temporal pattern, and spike latency codes, and argues that multiple coding strategies often coexist within sensory systems (Kamaleddin, 2022). They further show how temporal coding enables multiplexing, allowing single neural populations to encode multiple stimulus attributes simultaneously across different time scales. By synthesizing evidence across modalities, their contribution positions multiplexed encoding as a fundamental and ubiquitous principle of neural computation. Taken together, these studies bridge levels of analysis, from circuit-level gating and temporal coding mechanisms to system-level representations and computational implementations, providing a unified perspective on how multiplexed neural coding operates across biological and artificial domains (Kamaleddin, 2026).

Collectively, these findings underscore a recurring theme: multiplexing in neural coding is achieved through both circuit-level interactions and temporal dynamics. In biological systems, inhibitory gating and state-dependent modulation allow the same input to support multiple behavioral interpretations. In artificial neuromorphic systems, temporal coding strategies enable efficient representation of multiple stimulus features within sparse spike trains. These parallels suggest that biological inspiration remains a powerful guide for designing energy-efficient and adaptive computational models.

Extending beyond sensory pathways, this Research Topic also includes work on neural decoding in brain–computer interfaces, illustrating how principles of adaptive and context-aware coding generalize to higher-level neural signals. Wang et al. introduce a task-conditioned prompt learning framework for motor imagery EEG decoding, addressing the challenge of inter-subject variability. By encoding subject-specific information as prompt tokens that modulate a shared model, the approach enables rapid adaptation from limited data. This strategy reflects a broader shift toward parameter-efficient personalization, in which neural representations are dynamically conditioned rather than fully retrained (Lotte et al., 2018). Conceptually, this mirrors sensory polysemia: the same underlying representation can yield different outputs depending on contextual “prompts,” whether arising from internal states in biological systems or learned embeddings in artificial networks.

Across these diverse contributions, several unifying insights emerge. First, context is not an external modifier but an integral component of neural coding. Through inhibitory gating, temporal dynamics, and learned conditioning mechanisms, neural systems continuously integrate internal and external variables to shape perception and action. Second, multiplexing is a fundamental strategy for efficient information processing, enabling multiple stimulus attributes or interpretations to coexist within shared neural substrates. Third, biologically inspired principles such as sparsity, temporal coding, and inhibitory modulation provide valuable design constraints for artificial systems, particularly in domains requiring low power consumption and real-time processing.

At the same time, important challenges remain. The interplay between different coding schemes, such as rate-based, temporal, and population codes, requires further clarification, particularly in naturalistic settings where noise, variability, and behavioral context interact (Kamaleddin, 2025; Kamaleddin et al., 2022). Additionally, bridging scales from single-neuron dynamics to large-scale network function continues to be a critical frontier. Finally, translating insights from controlled experimental paradigms to real-world applications, including robotics and clinical neurotechnology, will require integrating robustness, adaptability, and interpretability.

Moving beyond static and unidimensional representations, the field increasingly recognizes that sensory and neural signals are dynamically interpreted, contextually modulated, and multiplexed across dimensions. By combining theoretical frameworks, computational models, and applied methodologies, the contributions presented here provide a more unified account of how neural systems encode information and how these principles can be leveraged in next-generation intelligent systems.
